# Development and Validation of Comprehensive Healthcare Providers’ Opinions, Preferences, and Attitudes towards Deprescribing (CHOPPED Questionnaire)

**DOI:** 10.3390/pharmacy10040076

**Published:** 2022-07-01

**Authors:** Iva Bužančić, Maja Ortner Hadžiabdić

**Affiliations:** 1 Faculty of Pharmacy and Biochemistry, University of Zagreb, A. Kovačića 1, 10 000 Zagreb, Croatia; mortner@pharma.hr; 2 City Pharmacies Zagreb, Kralja Držislava 6, 10 000 Zagreb, Croatia

**Keywords:** deprescribing, questionnaire, barriers, facilitators, primary care

## Abstract

Successful implementation of deprescribing requires exploring healthcare professionals’ opinions, preferences, and attitudes towards deprescribing. The aim of this study was to develop and validate the questionnaire exploring healthcare providers’ opinions preferences and attitudes towards deprescribing (CHOPPED questionnaire). This was a cross-sectional on-line survey. A comprehensive 58-item questionnaire, in two versions (for pharmacists and physicians), was developed through an extensive literature review and interviews with experts. The questionnaire was validated, and its reliability was assessed through data collected from 356 pharmacists and 109 physicians. Exploratory factor analysis was performed, and 37- and 35-item questionnaires were developed. Ten factors were identified: knowledge, awareness, patient barriers and facilitators, competencies barriers and facilitators, collaboration barriers and facilitators, and healthcare system barriers and facilitators. The CHOPPED tool has satisfactory face, content (CVR > 0.62) (content validity ratio), construct, and criterion validity. The reliability statistics of all factors in both versions was acceptable with Cronbach’s alpha > 0.6. Test–retest reliability analysis showed that gamma rank correlations of total factor scores were strong and very strong (between 0.519 and 0.938). The CHOPPED tool can be used as a valid and reliable tool to explore healthcare providers’ opinions and attitudes toward discontinuing medications in the primary care setting in Croatia.

## 1. Introduction

Increasing life expectancy of populations and availability of medical care lead to inappropriate prescribing, polypharmacy, and poor health outcomes, especially in the elderly [1,2,3]. Healthcare practitioners and researchers have many tools available to combat this ever-growing problem, one of them being deprescribing. Deprescribing can be described as an essential part of prescribing and involves identifying inappropriate medicines that should be reduced or discontinued as their continuing use brings more harm than benefit to the patient [4]. Research shows that most problems in deprescribing arise from a lack of well-established and implemented services in standard practice. Even though deprescribing as a clinical intervention has been in focus in the past decade and many feasibility trials and protocols have been developed, there is still a lack of implementational studies and strategies. Several authors and publications have accentuated this particular problem and declared it as a priority in future research [5,6,7]. Ailabouni and co-authors in their commentary from 2022 address the current limitations of implementing deprescribing guidelines into practice and policy, and how implementation science can be of service [8].

Deprescribing requires thoughtful engagement of all stakeholders, patients, and their healthcare providers, including physicians, specialist doctors, and pharmacists. The introduction and implementation of deprescribing in everyday clinical practice, for both primary care physicians and community pharmacists, are demanding and are influenced by many factors. While patient characteristics might be considered most important, a number of qualitative health research studies show a variety of barriers for healthcare providers to identify when considering deprescribing [9]. In each healthcare system, unique determinants that influence the ability to provide deprescribing can be identified. Gathering information and determining factors from first-line healthcare providers in those systems can aid in creating implementational strategies [8,10].

Qualitative research is valuable in identifying concepts and themes of a new service but is often limited by the number of participants or excludes participants less familiar with the topic. Most commonly identified themes or concepts include patient expectations, medical culture, fear of damaged reputation, ethical, legal, and financial dilemmas, lack of organization, uncertainty in skills or abilities and professional identity, and access to information to name a few [7,11,12,13,14]. To reach a larger and more diverse population of healthcare providers, surveys can be used. Until recently, only a few attempts were made to develop and validate a tool that could explore barriers and facilitators of deprescribing. Linsky et al. developed an instrument valuable for exploring prescribers’ perceptions of medication discontinuation [15]. The instrument was used on healthcare providers familiar and versed in deprescribing. For healthcare systems with developing pharmaceutical care, as well as for different levels or settings within a healthcare system unfamiliar with deprescribing, it is essential to explore healthcare providers’ perceived barriers and enablers of deprescribing to ensure successful implementation of a novel service [5]. Therefore, the aim of this study is to develop and validate a Comprehensive Healthcare providers’ OPinions, Preferences, and attitudEs towards Deprescribing questionnaire (CHOPPED questionnaire). The questionnaire is developed considering both prescribers and those without prescribing benefits as their viewpoints might differ, as well as those who do not provide deprescribing as standard practice.

## 2. Materials and Methods

### 2.1. Design

A cross-sectional survey was conducted on registered community pharmacists and primary care physicians in Croatia.

#### 2.1.1. Development of the CHOPPED Questionnaire

For item development, both deductive and inductive methods were used [16]. An extensive literature examination was performed, including qualitative design studies, commentaries, letters to the editors, expert opinions, and systematic reviews on the topic, in order to identify key concepts, themes, and factors [9,14,15,17,18,19,20,21,22,23,24,25,26,27]. Authors identified three frequently appearing themes: patient, profession, and organisation. For each theme, the most commonly occurring concepts were systematized. These included professional accountability, system support, communication, finance and legal, prescribing, patient wishes and desires, beliefs about medication appropriateness and harm, and relationships and perceptions. One-on-one interviews with ten primary health care providers (six pharmacist and four physicians) were conducted on the topic of medication stopping, and potential obstacles and motivators needed for providing such a service were identified. To help guide the interviews for each concept, researchers formulated prompts (Table 1). Involved healthcare providers came from different clinical backgrounds and had diverse and complementary skills in pharmaceutical care. During interviews, researcher eliminated prompts considered unnecessary or those that were not mentioned by the interviewees and formulated preliminary items. Highly similar items were then merged or removed. Based on interview data and proposed themes and domains, a comprehensive 58-item questionnaire was prepared by two researchers, in two versions, one for physicians and one for pharmacists (Supplementary File S1) All questions from this part of the questionnaire used a 5-point Likert scale (1 = strongly disagree, 2 = disagree, 3 = neither agree nor disagree, 4 = agree, 5 = strongly agree) as possible answers.

The CHOPPED questionnaire was further extended by the inclusion of a case vignette based on a real-life patient. The case vignette was intended to assess pharmacists’ and physicians’ agreement on deprescribing decisions (File S2). In the pharmacists’ version, respondents had to indicate which medication they would suggest for deprescribing and state the rationale behind their answer. In the physicians’ version, respondents had to indicate which pharmacist deprescribing suggestion they would agree with. The patient in the vignette was a community-dwelling older adult with 15 medications, four comorbidities, low activity of the daily living score, and high willingness to have medication deprescribed.

#### 2.1.2. Participants

LIMESurvey^®^ software was used to design and distribute the questionnaire. Dillman’s guiding principles for mail and internet surveys were used to help with the survey design [28]. The survey was sent via email to available community pharmacies and physicians’ practices from the directory of the health insurance fund and professional affiliations (national chamber of pharmacists and national chamber of physicians). In the invitation email, potential participants were asked to forward the link to the survey to fellow pharmacists or physicians. Two email reminders, four and eight weeks after the initial email, were sent. All responses were anonymous. Informed consent was included in the survey and was set as a required response to ensure all participants were informed on all aspects of the study. Potential participants who did not digitally authorise the informed consent could not access the survey. The study was conducted between October 2021 and January 2022 in Croatia. It was approved by the Ethics Committees of City pharmacies Zagreb and Health Centre Zagreb. Participants could save the answers of the unfinished questionnaire and complete the questionnaire at a later time. To ensure there were no duplicate inputs, each unique IP address was marked in the responses. If a single IP address had multiple inputs, cross-checking was performed for socio-demographic information. Duplicate unfinished or answerless questionnaire entries from duplicate IP addresses were discarded. Participants’ inputs were included in the validation analysis if all of the questions were answered.

As literature reports for adequate sample size for validity analysis vary, a general rule of subject to item ratio of 2:1 to 10:1 was considered, and 4:1 ratio was employed [29,30]. For test–retest reliability, a sample size of 20 was chosen, and for exploratory factor analysis, a sample size of 200 was considered adequate [31]. The response rate was expected to be around 20%; therefore, the questionnaire was sent to at least 1000 email addresses.

### 2.2. Validation

Face validity, content validity, construct validity, criterion validity, internal consistency, and test–retest reliability were the chosen methods of validation.

#### 2.2.1. Face Validity

Healthcare providers involved in item generation, described above, participated in the face validity assessment. They were invited to a group discussion to review and comment on prepared preliminary versions of the questionnaire. Each item was examined and rated on clarity, relevance, and importance. Finally, panellists assessed whether appropriate phrasing was used, and necessary changes were made before validation and widespread administration.

#### 2.2.2. Construct Validity

Exploratory factor analysis (EFA) was performed to identify factors and to refine the questionnaire length [32]. The analysis was performed concurrently for both versions of the questionnaire. Promax rotation was chosen as a rotation method of EFA since there was correlation between factors. The criteria to retain the number of factors included the eigenvalue, the scree plot test, the proportion of total variance accounted for, and the interpretability criterion. The eigenvalue signifies the amount of variance in all of the values for which the factor accounts for. A value > 1 implies that the factor accounts for more variance than an average single item [33]. For the proportion of the total variance, factors explaining 60% to 70% of total variance were retained. Sampling adequacy was confirmed using the Kaiser–Meyer–Olkin statistic and the suitability for reduction using the Bartlett’s test of sphericity. Additionally, parallel analysis was used to compare and confirm proposed number of factors. During EFA item reduction was performed as well, analysing inter-item and item-total correlations. Items with loading value <0.3 and those loaded on two or more factors (>0.32) were removed first. Before item removal the research team discussed the potential impact of the item, and the final decision was made based on reached consensus. Several variations of the EFA were performed to ensure the best possible combination of items formed the final versions of the questionnaire (considering loading values, variance, internal consistency, and practical matters). The model was considered having a good fit if less than 50% of the non-redundant residuals had absolute values >0.05. The pharmacists’ data was randomly split into two groups (60% and 40% of participants to ensure adequate sample size) and the factors were developed on the 60% of the data. To confirm the proposed factors, 40% of pharmacists’ data was used and forced factors extraction method was utilized. Physicians’ data was insufficient to split and repeat the EFA.

#### 2.2.3. Content Validity

Content validity was assessed employing Lawshe’s method [34]. A panel of ten healthcare providers, which have not been involved in item development, scored post-EFA items as ‘’essential’’, ‘’useful but not essential’’, and ‘’not necessary’’. For each item content validity ratio (CVR) was calculated. Responses ‘’essential’’ and ‘’useful but not essential’’ were combined. Items with CVR < 0.62 (value of at least 0.62 equals > 80% of panellists considering the item to be essential or useful but not essential) were reviewed and removed.

#### 2.2.4. Scoring of the Questionnaire

As a 5-point Likert scale was used the following scoring system was proposed: each item could be scored from 1 to 5. Factor score was calculated by summing all item scores and averaging with the number of items in that factor. That way each factor could have a score from 1 to 5. The overall factor theme determined the direction of scoring.

#### 2.2.5. Criterion Validity

Criterion validity was established exploring this proposed hypothesis: items or factors associated with higher positivity towards deprescribing will be correlated with higher knowledge and awareness about deprescribing. Factors associated with obstacles towards deprescribing will be negatively correlated with willingness to suggest deprescribing, while facilitators of deprescribing will be positively correlated willingness to suggest deprescribing.

#### 2.2.6. Reliability

The reliability of the final versions of questionnaire was assessed by determining internal consistency of the questionnaire and performing a test–retest. Internal consistency was determined for each factor via Cronbach’s alpha testing. Items that increased Cronbach’s alpha when deleted were removed from the questionnaire. For test–retest reliability new twenty healthcare providers were recruited. They were given hard copies of the final versions of the questionnaire to complete. Retest was scheduled two weeks later. Test–retest reliability of individual items was determined using linear-weighted Cohen’s kappa. A Cohen’s kappa coefficient of <0.20 was considered poor, 0.2–0.4 fair, 0.41–0.60 moderate, 0.61–0.80 good, and >0.80 very good [35]. Gamma rank correlation was used to determine the test–retest reliability of factor scores.

### 2.3. Case Vignette

The case was reviewed by a clinical pharmacy specialist and academic researcher to ensure proposed answers agreed with available guidelines in prescribing and deprescribing of potentially inappropriate medications.

### 2.4. Data Analysis

Sociodemographic data was analysed using descriptive statistics. For all analyses, a value of *p* < 0.05 was considered statistically significant. All analyses were performed using IBM SPSS Statistics for Windows, Version 26.0. (IBM Corp., Armonk, NY, USA).

## 3. Results

### 3.1. Participants

**Figure 1:**
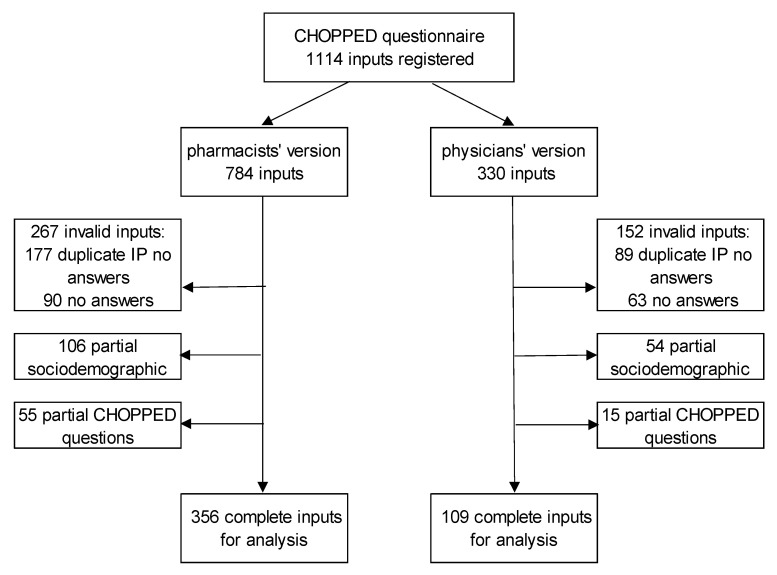
Participant recruitment and data selection.

Pharmacists’ version of the CHOPPED questionnaire was sent to 1108 email addresses, and physicians’ version was sent to 773 addresses. Response rate could not be estimated as there was no way to collect the precise number of email addresses the survey was sent to via the snowball sampling. For pharmacists’ version 784 inputs were registered and for physicans’ version 330. Overall, 356 pharmacists’ and 109 physicians’ complete inputs were available for validation analysis (Figure 1). Pharmacist who provided sociodemographic information but did not complete the survey were not statistically different in terms of age, years of experience, level of educational attainment or type of pharmacy practice to those who completed the survey. There was no statistically significant difference in characteristics (age, years of experience, practice characteristics, number of patients, or number of elderly patients in practice) among physicians who completed the survey and those who did not.

Both pharmacists and physicians were mostly female (86.23% pharmacists and 68.80% physicians). Pharmacists had a median age of 35 years (IQR 28–43), a median professional experience of 10 years (IQR 3–19.75) while physicians had a median age of 51 (IQR 33–59), a median professional experience of 23 years (IQR 7–31.50). Majority of healthcare providers worked in an urban area (58.43% pharmacists and 42.20% physicians) (Table 2) Based on surveys time stamp, the median time to complete the first part of the questionnaire was 8 min (IQR 3–16) for both pharmacists and physicians. Pharmacists spent a median of 16 min (IQR 5–24) completing the case vignette, while physicians spent a median 4 min (IQR 0.5–12).

### 3.2. Questionnaire Validation and Item Reduction

#### 3.2.1. Construct Validity

The sampling adequacy of the 60% of the pharmacists’ sample (*n* = 214) was confirmed using the Kaiser–Meyer–Olkin measure (0.834); Bartlett’s test of sphericity (*p* < 0.001) confirmed the factorability. The scree plot indicated 12 factors that accounted for 40.67% of variance when all items were used in the analysis. After final extraction analysis, 37 items were retained and grouped into 10 factors. The final 10 factors accounted for 53.87% of the variance. There were 3% nonredundant residuals with absolute values greater than 0.05. When repeating the EFA on the remaining 40% of the sample (*n* = 142) and using the forced factor extraction, all items loaded on the same factors. Five questions showed cross-loading with respect to other factors (loading values < 0.3200) but loaded the most strongly on the original factor. Parallel analysis confirmed 10 factors.

For the physicians’ version of the questionnaire, the Kaiser–Meyer–Olkin measure of sampling adequacy was 0.759, and Bartlett’s test of sphericity (*p* < 0.001) confirmed the factorability. The scree plot indicated 12 to 14 factors explaining 43.27% and 46.48% of the variance when all items were analysed. Several models were explored by removing and adding items in the analysis to achieve factors similar to those in the pharmacists’ version. Finally, 35 items formed 10 factors, which accounted for 58.07% of the variance. The 10 factors were equal to those in the pharmacists’ version and were confirmed with parallel analysis. There were 7% nonredundant residuals with absolute values greater than 0.05. There were certain differences in item loadings in the two versions. Questions, “*I worry that stopping medications could lead to adverse drug withdrawal effects or worsening of patient’s health*”, “*A decision support tool within healthcare providers software would enable me to suggest stopping medications more*”, and “*I believe there is a disproportion between available guidelines on prescribing and stopping medications which makes it difficult for me to suggest deprescribing*” did not significantly load on any factors in the physicians’ version of the questionnaire. Questions “*Having the possibility to contact a task force or a professional network when I am having doubts regarding stopping or reducing medications, would encourage me to suggest such changes*” and “*Lack of direct in-real-time communication with other healthcare providers (hospital doctors or specialists, pharmacist, nursing home care teams...) makes it difficult for me to suggest stopping or reducing medications*” did not significantly load on any factors in the pharmacists’ version.

The retained 10 factors were grouped into three domains best described as: Knowledge and awareness about deprescribing, Barriers to deprescribing, and Facilitators of deprescribing. Knowledge and awareness about deprescribing contain seven items in two factors. In Barriers to deprescribing and Facilitators of deprescribing, four factors can be identified: patient factor, competencies factor, collaboration factor, and healthcare system factor. Each factor was explored with three or four items (Table 3).

The question “*I am willing to suggest deprescribing to a patient if appropriate*” was removed during EFA as it did not significantly load on any factor but was retained in both final versions of the questionnaire due to its overall importance.

Two versions of the questionnaire and differences in items can be seen in Table 3, and the source of questions and the initial version of the questionnaire can be seen in File S1.

#### 3.2.2. Content Validity

During the content validity assessment, the CVR for all items was calculated as >0.62, and as such, no items were removed (Table S1). The majority of items had a CVR of 1, and around 36% of items had a CVR of 0.8.

#### 3.2.3. Scoring of the Questionnaire

A simple unweighted approach was chosen. Factors were scored so that the higher score indicated higher knowledge and awareness of deprescribing, as well as a greater effect of barriers or facilitators on deprescribing. Willingness to deprescribe was not scored, and a total score for the complete questionnaire was not developed.

In the pharmacists’ version, the mean value of factor scores was knowledge 4.04 ± 0.88, awareness 4.57 ± 0.57, patient facilitator 3.63 ± 0.81, collaboration facilitator 4.51 ± 0.58, competencies facilitator 4.45 ± 0.65, healthcare system facilitator 4.22 ± 0.78, patient barrier 3.21 ± 0.72, collaboration barrier 3.65 ± 1.01, competencies barrier 3.42 ± 0.74, and healthcare system barrier 3.89 ± 0.75. In the physicians’ version, the mean value of factor scores was knowledge 3.71 ± 0.84, awareness 4.19 ± 0.77, patient facilitator 3.77 ± 0.67, collaboration facilitator 3.78 ± 0.93, competencies facilitator 3.95 ± 0.84, healthcare system facilitator 3.90 ± 0.80, patient barrier 3.11 ± 0.81, collaboration barrier 3.16 ± 0.76, competencies barrier 2.87 ± 0.82, and healthcare system barrier 3.84 ± 0.81. A detailed table with minimum and maximum values can be found in Table S2.

#### 3.2.4. Criterion Validity

Greater knowledge and awareness were correlated with greater willingness to suggest deprescribing in both pharmacists’ and physicians’ data (G = 0.228; *p* < 0.001 and G = 0.292; *p* = 0.002, respectively). In the pharmacists’ data, an increased perception of barriers of deprescribing was inversely correlated with the willingness to suggest deprescribing to patients (G = −0.182, *p* = 0.001). Facilitators of deprescribing were statistically significantly associated with the willingness to suggest deprescribing (G = 0.298, *p* < 0.001). In the physicians’ data, a greater perception of facilitators of the deprescribing score was associated with greater willingness to suggest deprescribing (G = 0.213, *p* = 0.026). There was no correlation between physicians’ willingness to deprescribe and barriers (G = 0.115, *p* = 0.193).

#### 3.2.5. Reliability

Internal consistency was assessed by analysing Cronbach’s alpha, which showed satisfactory scores for all factors in both versions of the questionnaire [36]. In the pharmacists’ version, competencies facilitators and the collaboration barriers had exemplary internal consistency (>0.8), while awareness, patient facilitators, collaboration facilitators, and competencies barriers had extensive internal consistency (>0.7). Internal consistency for knowledge, healthcare system facilitators, patient barriers, and healthcare system barriers was moderate (>0.6) [37]. In the physicians’ version, awareness and competencies facilitators had exemplary internal consistency (>0.8). Knowledge, patient facilitators, collaboration facilitators, patient barriers, and healthcare system barriers had extensive internal consistency (>0.7). Moderate internal consistency was found for healthcare system facilitators, collaboration barriers, and competencies barriers, with Cronbach’s alpha levels >0.6 (Table 1). No individual item of each factor increased the alpha score when deleted; therefore, no item was deleted for the respective factors.

Repeatability based on the analysed linear-weighted Cohen’s kappa was fair for two questions, moderate for 36, good for 14, and very good for four questions (Table 3). Gamma rank correlations of total factor scores were strong and very strong. In the pharmacists’ version, the knowledge factor had a G value of 0.938, the awareness factor had G = 0.519, patients’ facilitators had G = 0.552, collaboration facilitators had G = 0.929, competencies facilitators had G = 0.531, and the healthcare systems facilitators had G = 0.881. In the barriers theme, patients’ barriers had G = 0.826, collaboration barriers had G = 0.544, competencies barriers had G = 0.789, and healthcare system barriers had G = 0.645. In the physicians’ version, the G values were as follows: knowledge factor 0.864, awareness factor 0.565, patients’ facilitators 0.695, collaboration facilitators 0.812, competencies facilitators 0.559, healthcare system facilitators 0.844, patients’ barriers 0.902, collaboration barriers 0.623, competencies barriers 0.682, and healthcare systems’ barriers 0.545. All factor score correlations were statistically significant, with *p* < 0.001.

## 4. Discussion

This study describes the development and validation of a novel tool that can explore healthcare providers’ opinions and preferences regarding deprescribing. Validation analysis demonstrated that a psychometrically rational questionnaire was developed. Factors such as knowledge and awareness, as well as facilitator factors, were correlated with a greater willingness to suggest deprescribing. At the moment, there are several attempts in the development of a questionnaire suitable for healthcare providers [15,38,39,40]. This accentuates the global need for tools to widely investigate important deprescribing factors that might influence implementation in everyday practice. The tool developed by Shrestha and colleagues, designed to explore healthcare professionals’ attitudes towards deprescribing in older adults with limited life expectancy (HATD tool), identified factors named concerns and assurance, which have a similar construct to the CHOPPED competencies barriers and facilitators [40]. The Brazilian Desmedica Study protocol describes a conceptual framework with similar themes such as the health system or patient context [38]. These similarities additionally confirm not only universally recognized barriers, but also that the CHOPPED questionnaires’ factors have the potential to be applied to different healthcare systems around the world. The questionnaire proposed in this study is one of the first tools to be validated and used amongst healthcare providers inexperienced in deprescribing. The tool explores general barriers and facilitators, regardless of the type of patient or medication aimed to deprescribe. The CHOPPED questionnaire contains 10 factors meaningful for potential deprescribing. Adequate knowledge and awareness of the benefits of a service, such as deprescribing, are an important basis for future implementation [41]. An extensive literature review helped to generate potential questionnaire items, showing the complex background of deprescribing, as well as the impact it has on healthcare providers’ attitudes [6,13,14,19,23,25,42,43,44,45,46]. This questionnaire aimed to quantitatively capture these barriers and facilitators. Patient factor items explore the connection between healthcare providers’ willingness and hesitancies to deprescribe and a patient’s involvement with medication, as well as the influence of deprescribing on their relationship. Items from the collaboration factors explore how inter- and intra-professional collaboration can affect potential deprescribing decisions. The competencies factors examine healthcare providers’ necessary skills and intrinsic motivation needed to suggest deprescribing. Healthcare system factors explore how policy, legislation, renumeration, access to information, or workplace organization affect deprescribing initiatives. Certain similar concepts were explored in qualitative studies [47,48,49]. Item: ’’I am willing to suggest deprescribing to a patient if appropriate’’ was kept in the questionnaire even though it did not load significantly on any factor. It was deemed to be essential by all the panellists as it could potentially quantitatively define the true willingness to suggest deprescribing and could potentially be correlated with the suggested deprescribing factors.

A case vignette ensured participants were given a clinical conundrum similar to those seen in everyday practice. Case vignettes can be a useful learning and implementational tool, but a detailed analysis was out of scope for this manuscript [50,51]. Future in-depth analysis of the case vignette answers could be correlated with the questionnaire factor scores. This could help outline types of healthcare providers who are keener to suggest deprescribing. It can also be used to identify common deprescribing misconceptions.

Two versions were developed, one for pharmacists and one for physicians. This, alongside a case vignette, additionally distinguishes CHOPPED from other tools. One tool enables easier identification of common barriers and facilitators within the same healthcare system, while two versions allow finer understanding of professional specific viewpoints. The majority of the developed items (regarding patients, competencies, or healthcare systems) were appropriate for both pharmacists and physicians. When it came to differences between professions or their responsibilities, equivalent items were developed. For example, items regarding collaboration were formulated from the position of a certain healthcare provider. The main item of willingness to suggest deprescribing remained the same for both professions. Deprescribing is first and foremost a patient-centred process, and each different healthcare provider can substantially contribute to effective deprescribing. Focus groups, semi-structured interviews, and case vignettes are the most commonly used research methods, and general practitioners and healthcare providers with prescribing privileges are the most common research participants [11,26,27,52,53,54,55,56]. Healthcare systems are recognizing pharmacists as valuable deprescribing partners, and research shows pharmacist-led or collaborative deprescribing interventions are effective and safe [57,58,59,60,61]. Examining profession-specific viewpoints can be beneficial for achieving the multi-disciplinary approach deprescribing requires. The CHOPPED questionnaire has the potential to be used in primary care settings where other healthcare providers, such as nurses (nurse practitioners’ practices, mobile nursing practices), have prescribing rights or participate in deprescribing. Recently, there has been a surge of research publications regarding nurses’ positions and perspectives in deprescribing, especially in terms of geriatric patients [62,63,64]. Depending on the particular nursing professionals’ responsibilities, both versions of CHOPPED could be used. The pharmacists’ version of the questionnaire with minor changes might be a good starting point to explore nurses’ perceptions of deprescribing, especially for those without prescribing rights. For those with prescribing rights, the adjusted physicians’ version could be used. In healthcare systems with a GP-nurse–pharmacist team care for the same patient, it would be prudent to educate and involve nurses in deprescribing as well.

Analysis showed that the developed tool has satisfactory face, construct, and content validity for both versions. Criterion validity was established for both versions as well, but additional research is needed to confirm other types of criterion validity, especially concurrent validity using other scales.

### Strengths and Limitations

The test–retest subjects had a median age of 33 years (IQR 26–40), and a median of seven years of professional experience (IQR 2–14), being somewhat younger than the participants in the validation samples (Additional information on test–retest participants’ characteristics is provided in Supplementary Table S3). This could have influenced the values of the reliability analysis. Lower Cohen’s kappa for certain items might be due to changes in perception of items in the questionnaire as well as changes in knowledge and opinion when retesting. Deprescribing is a relatively new topic amongst the test–retest participants. There was no significant difference in the test–retest scores between the two professions. The range of scores implies the scales have the ability to capture differences in opinion and could indicate that participants have not merely provided a satisfactory answer. Moderate internal consistency was found for four factors in the pharmacists’ version and for three factors in the physicians’ version. Regardless, reliability analysis showed satisfactory internal consistency and adequate repeatability.

Additional limitation could concern the participants in the validation samples. A less age-diverse sample of pharmacists completed the questionnaire compared to physicians. Potential reasons could include younger participants having higher computer literacy or being more prone to using internet tools. During questionnaire development, several panellists commented on the possibility to distribute the survey in paper form, as many mature healthcare providers still prefer such surveys. Due to the COVID-19 pandemic and a lack of face-to-face events, where such a method could be used, it was viewed that the internet distribution was a more wholesome option as it could reach healthcare providers in displaced and rural areas as well. More mature pharmacists could have had different opinions, which could have affected the correlation analysis. Furthermore, the physicians’ sample was three times smaller than the pharmacists’ sample, and a larger percentage of physicians’ inputs was invalid. The reasons for this lower participation rate could include a lack of time or interest in participation due to the pandemic. Further studies should include more experienced healthcare providers or different distribution methods to gain a more in-depth view of the topic. Most participants in both samples were female, which might be viewed as a limitation as well. Based on data from the Croatian Institute of Public Health and Croatian chamber of pharmacists, more than 60% of all physicians are female and more than 80% of all pharmacists are female [65,66]. The sample of healthcare providers in this study adequately represents the population of healthcare providers in Croatia.

While there is substantial qualitative research regarding barriers and facilitators of deprescribing [67], this study brings a novel tool that can be used in different healthcare systems and in different levels of healthcare. Underdeveloped or developing healthcare systems are confronted with different barriers than developed healthcare systems with implemented and widely accepted pharmacists’ interventions. Gaining knowledge on potential barriers or facilitators can help such a system in policy and legislation development and in finding the best implementation strategy of a service such as deprescribing. For instance, a healthcare system’s barriers in certain settings might be greater than competencies barriers and could indicate changes in information access or an increase in personnel is needed.

The length of the questionnaire could be viewed as a potential limitation. It was developed and validated on a population of healthcare providers new to deprescribing. The main aim of the CHOPPED questionnaire is to comprehensively and thoroughly explore all latent traits connected to deprescribing. Awareness of deprescribing was correlated with willingness to deprescribe. Raising awareness amongst inexperienced healthcare providers could potentially initiate deprescribing engagement. For healthcare providers familiar with deprescribing, only barrier and facilitator factors can be used. It would be beneficial to use the tool for such healthcare providers and compare and contrast barriers and facilitators.

The CHOPPED questionnaire has the potential to be universally used in the primary care setting. As evidence on deprescribing is growing, future revision of the questionnaire will most likely be necessary. Future research should include using the suggested questionnaire or its factors as a part of a deprescribing intervention.

## 5. Conclusions

A comprehensive questionnaire exploring healthcare providers’ attitudes towards deprescribing was developed. Ten factors were identified: knowledge, awareness, patient barriers and facilitators, competencies barriers and facilitators, collaboration barriers and facilitators, and healthcare systems’ barriers and facilitators. The tool has the potential to help identify obstacles and enablers of deprescribing in the primary care setting and facilitate implementation of the deprescribing process.

## Figures and Tables

**Table 1:** Themes, concepts, and prompts used to develop the questionnaire items.

Patient		Profession		Organization
*Wishes and desires* burden desire to stop involvement duration of pharmacotherapy health perception wellbeing expectations resistance to change candidates for medication review candidates for medication discontinuation	*Prescribing* pressure to prescribe pressure to dispense reluctance to change medications justification of illness overprescribing overconsumption	*Professional accountability* knowledge opinion capabilities and confidence opportunities to act indifference uselessness fear or hesitation	*Communication* fragmentation of care access to information transfer of care	*System support* time space guidelines system alerts education technology utilization
*Relationships and perceptions* loss of trust feeling of abandonment shared decision making prior positive experience positions of authority	*Beliefs about medication appropriateness and harm* better safe than sorry attitude side effects end of life comfort	*Relationships and perceptions* inter-professional intra-professional professional courtesy division of responsibilities shared-decision making hierarchy		*Finance and legal* reimbursement penalties repercussions ethics policy

**Table 2:** Participant characteristics.

Characteristics	Pharmacists	Physicians
Sex female (n, %)	307 (86.23%)	75 (68.80%)
Age (median, IQR)	35 (28–43)	51 (33–59)
Professional experience (median, IQR)	10 (3–19.75)	23 (7–31.5)
Practice location (n, %)		
urban	208 (58.43%)	46 (42.20%)
suburban	114 (32.02%)	38 (34.86%)
rural	34 (9.55%)	25 (22.93%)
Practice placement (n, %)		
within another healthcare facility	61 (17.13%)	75 (68.81%)
near another healthcare facility	140 (39.33%)	N/A
within a shopping facility	19 (5.34%)	N/A
displaced (not near any facility)	136 (38.20%)	34 (31.19%)
Patient population (median, IQR)		
number of patients	N/A	1600 (1216–1860)
percentage of elderly patients (>65 years)	N/A	35% (28.50–47.50)

**Table 3:** Questionnaire validation and reliability analysis.

Factor	Item	Factor Loading			Item: Total Correlation		Test–Retest Reliability ^c^	
		Pharmacists’Version Development	Pharmacists’ Version Repeatability	Physicians’ Version	Pharmacists’ Version	Physicians’ Version	Pharmacists’ Version	Physicians’ Version
Knowledge factor Cronbach’s α 0.684/0.703	tapering or reducing a dose	0.707	0.894	0.421	0.519	0.339	0.85	0.74
	changing medication to a safer alternative	0.720	0.543	0.633	0.519	0.339	0.47	0.57
	method of discontinuing a drug	0.624	0.540	0.671	0.505	0.541	0.59	0.52
Awareness factorCronbach’s α 0.783/0.811	important as prescribing medication	0.378	0.786	0.769	0.505	0.665	0.38	0.61
	reduces health care expenditures/costs	0.707	0.735	0.549	0.644	0.472	0.36	0.43
	improve patient adherence	0.793	0.719	0.808	0.596	0.721	0.51	0.51
	patient outcomes	0.771	0.434	0.764	0.561	0.654	0.88	0.80
Patient Facilitators factorCronbach’s α 0.776/0.713	patient desire to reduce	0.852	0.697	0.646	0.585	0.580	0.61	0.49
	successful prior stopping of medication	0.818	0.788	0.839	0.654	0.624	0.25	0.29
	easily available patient materials	0.436	0.718	^b^	0.501	^b^	0.51	^b^
	patients with greater involvement	0.571	0.798	0.361	0.611	0.585	0.44	0.39
Collaboration Facilitators factorCronbach’s α 0.787/0.744	collaboration with pharmacist//collaboration with physician	0.572	0.506	0.730	0.542	0.558	0.48	0.48
	physicians contact pharmacists regarding patient care	0.589	0.428 ^a^	^b^	0.548	^b^	0.57	^b^
	evidence-based pharmacists’ rationale	^b^	^b^	0.803	^b^	0.641	^b^	0.59
	a public health project	0.808	0.378	0.854	0.649	0.763	0.49	0.36
Competencies Facilitators factorCronbach’s α 0.870/0.861	continuing education on the rationale	^b^	^b^	0.686	^b^	0.585	^b^	0.50
	guidelines or algorithms	0.562	0.600 ^a^	0.884	0.706	0.788	0.36	0.58
	how to approach patients	0.824	0.884	0.925	0.746	0.804	0.36	0.70
	medication review and management	0.682	0.884	0.872	0.713	0.801	0.47	0.69
	reminder/decision support tool	0.817	0.721	^b^	0.700	^b^	0.52	^b^
Healthcare systems Facilitators factorCronbach’s α 0.694/0.629	reimbursement	0.454	0.593	0.781	0.463	0.447	0.48	0.54
	contact a task force or a professional network	^b^	^b^	0.510	^b^	0.351	^b^	0.37
	patients’ medical records access	0.409	0.543	^b^	0.520	^b^	0.37	^b^
	additional staff members	0.591	0.441 ^a^	0.363	0.507	0.407	0.30	0.54
Patient Barriers factorCronbach’s α 0.668/0.730	patients using medications for a long time	0.684	0.564	0.697	0.486	0.465	0.86	0.53
	harm my relationship with my patient	0.600	0.624	0.448	0.436	0.502	0.64	0.62
	patients with low understanding of their therapy	0.482	0.540	0.604	0.390	0.509	0.64	0.55
	insisting on continuing prescribing	^b^	^b^	0.829	^b^	0.583	^b^	0.45
	adverse effects or worsening of patient’s health.	0.416	0.338	^b^	0.501	^b^	0.72	^b^
Collaboration Barriers factorCronbach’s α 0.899/0.635	pharmacists suggestions are inappropriate	0.652	0.632	0.736	0.644	0.553	0.40	0.36
	lack of direct communication	^b^	^b^	0.405	^b^	0.643	^b^	0.44
	negatively influence relationship with prescribers//inappropriate to stop medications prescribed by other physicians	0.816	0.873 ^a^	0.403	0.810	0.632	0.41	0.60
	physicians not understanding pharmacist//inappropriate for another physician to stop medications	0.843	0.926	0.442	0.823	0.515	0.50	0.34
	physicians find pharmacist unknowledgeable	0.940	0.865	^b^	0.825	^b^	0.67	^b^
Competencies barriers factor Cronbach’s α 0.713/0.687	unable to identify potentially inappropriate medicines	0.630	0.711	0.333	0.460	0.495	0.48	0.38
	lack of confidence	0.657	0.580	0.891	0.580	0.549	0.48	0.82
	disproportion of guidelines	0.609	0.481	^b^	0.479	^b^	0.37	^b^
	apprehensive to stop preventative medication.	0.470	0.518 ^a^	0.452	0.489	0.455	0.31	0.38
Healthcare systems Barriers factorCronbach’s α 0.642/0.741	lack of time	0.521	0.889	0.788	0.569	0.573	0.29	0.66
	additional documentation	0.455	0.442	0.797	0.383	0.501	0.22	0.53
	lack of space//fragmented patients care	0.480	0.442	0.527	0.558	0.481	0.52	0.52
	lack of policy and legislation	0.613	0.440	0.501	0.387	0.591	0.49	0.49
Willingness	willing to suggest deprescribing	NA	NA	NA	NA	NA	0.77	0.69

^a^ questions showing cross-loading with respect to other factors (loading values < 0.3200); loading was the most strongly related to the original factor, ^b^ questions not in the pharmacists’ or physicians’ version, ^c^ Cohen’s kappa.

## Data Availability

Not applicable.

## Supplementary Materials

The following supporting information can be downloaded at: https://www.mdpi.com/article/10.3390/pharmacy10040076/s1, File S1: The preliminary 58 items and source of items; File S2: CHOPPED case vignette; Table S1: Content validity assessment (number of panellist’s responses). Table S2: Distribution of factor scores; Table S3: Test–retest participants’ characteristics. References [68,69,70,71,72,73,74,75,76,77,78,79,80] are cited in the supplementary materials.
