# Supplementary material for: Development and Validation of Comprehensive Healthcare Providers’ Opinions, Preferences, and Attitudes towards Deprescribing (CHOPPED Questionnaire)
Source: Pharmacy (Basel). 2022 Jul 1;10(4):76. doi: 10.3390/pharmacy10040076 (PMC9326567; doi:10.3390/pharmacy10040076)
Supplement: Supplementary file 1 [file pharmacy-10-00076-s001.zip › file S1.pdf]

Development and Validation of Comprehensive Healthcare Providers’ Opinions, Preferences, and Attitudes towards Deprescribing (CHOPPED Questionnaire)

Iva Bužančić and Maja Ortner-Hadžiabdić

File S1: The preliminary 58 items and source of items

ITEM	SOURCE
Deprescribing is tapering or reducing a dose of a medication.	literature[1,2]
Deprescribing is changing medication to a safer alternative.	literature[2]
Deprescribing is a method of discontinuing a drug without a specific indication or benefit for a patient.	literature[2]
Deprescribing medication is as important as prescribing medication.	expert opinion
Deprescribing reduces health care costs.	expert opinion
Deprescribing can improve patient adherence.	literature[2], expert opinion
Deprescribing improves patient outcomes.	literature[3,4], expert opinion
Stopping medications should involve shared-decision making (process where the patient and healthcare provider work together to make health care decision that balances risks and expected outcomes with patient preferences and values).*	literature[5–8], expert opinion
Stopping medications is intended for/aimed at elderly patients with poor life expectancy.*	literature[2], expert opinion
Stopping medications is intended for patients with polypharmacy (5 or more medicines) regardless of their age.*	literature[2], expert opinion
Stopping medications is only intended for patients with side effects.*	focus group
Medication review and suggesting stopping medications should be performed when there is a clear indication for it (i.e., patient experiencing side effects, medicine unavailable, absence of ongoing symptoms).*	expert opinion
I often use tools like START/STOPP, Beers criteria, PRISCUS list, or PIMs lists when reviewing and dispensing medicines elderly patients use.*	expert opinion
Only patent’s primary prescriber can recommend stopping medication.*	focus group
I believe that suggesting stopping medications is a simple and easy task .*	expert opinion
I have confidence to suggest stopping medications.*	focus group
The decision which medications to stop should only be made by the prescriber.*	expert opinion, focus group
If a patient expressed their desire to have the number of their medication reduced, I would suggest stopping medications.	literature[5–8], focus group, expert opinion
A successful prior stopping of medication would encourage me suggest stopping medications to a patient again.	literature[9], focus group, expert opinion
Easily available patient education materials (brochures, booklets…) would help me educate and suggest stopping medications to my patients more.	focus group
I am keener to suggest stopping medications to patients who show greater involvement in their medication.	literature[6,9], focus group, expert opinion
I often discuss stopping medications with my patient.*	focus group
A close collaboration with a pharmacist who could provide patient follow-up during medication withdrawal would encourage me to suggest stopping medications more.	literature[6,10–12], focus group, expert opinion
A close collaboration with a physician to whom I could propose changes in pharmacotherapy would encourage me to suggest stopping medications more.	literature[13–15], focus group
I wish physicians would contact pharmacists more often when it comes to patient care.	focus group
Evidence-based rationale behind a pharmacist’s suggestion to stop medication would help me accept pharmacist’s suggestions.	literature[10,11,13,14], expert opinion
A public health project on deprescribing including a collaborative model (pharmacist and physicians) would encourage me to suggest deprescribing more.	literature[11], expert opinion
I feel I need more continuing education on the rationale behind stopping or reducing medication to be able to suggest such changes.	literature[4,6,8,15,16], focus group
I feel I need guidelines or algorithms to suggest stopping medications.	literature[4,9,15,17], focus group, expert opinion

A workshop or webinar on how to approach patients about stopping medication would help me suggest such changes more.	focus group
A course on medication review and medication management would help me suggest stopping or reducing medications more.	focus group
A decision support tool within healthcare providers software would enable me to suggest stopping medications more.	literature[3,7,9,16,18], focus group
I believe I need to be reimbursed for suggesting stopping medications as I am for dispensing/other clinical services I provide.	literature[5,6,11,13,16], focus group
Having the possibility to contact a task force or a professional network when I am having doubts regarding stopping or reducing medications, would encourage me to suggest such changes.	expert opinion, focus group
If I had the possibility to view patients' medical records (or parts of it important to pharmacist), I would be able to suggest stopping or reducing medications more.	literature[4,16], focus group
If I had additional staff members in my practice to take over a part of administrative workload, I would be able to suggest stopping or reducing medications more.	literature[6,16], focus group
I find it difficult to suggest stopping or reducing medications to patients who have been using them for a long time even if they no longer needed them.	literature[7], focus group
I am concerned that suggesting stopping medications could harm my relationship with my patient.	literature[9,11], focus group, expert opinion
I find it difficult to suggest stopping or reducing medications to patients who have low understanding of their therapy or to those that have low involvement in medication decision-making.	literature[7], focus group
Patient/patient's caregiver often insist I continue prescribing certain medication even if it is no longer necessary	literature[6,13,18], focus group
I worry that stopping medications could lead to adverse drug withdrawal effects or worsening of patient's health.	literature[6,8,13,19,20], expert opinion, focus group
I believe physicians find it inappropriate for pharmacists to suggest stopping or reducing medications. //<br>I find it inappropriate for pharmacists to suggest stopping or reducing medications to my patients.	literature[6,21,22], expert opinion
Lack of direct in-real-time communication with other healthcare providers (hospital doctors or specialists, pharmacist, nursing home care teams...) makes it difficult for me to suggest stopping or reducing medications.	literature[3,21,23], focus group, expert opinion
I worry that suggesting stopping or reducing medications could negatively influence my relationship with prescribers. //<br>I find it inappropriate to suggest stopping or reducing medications other physicians (other specialists) have prescribed.	literature[4,6,11,12,19,21], expert opinion
I worry physicians will not understand the rationale behind my suggestions. //<br>I find it inappropriate when another physician suggests stopping medications I have prescribed.	literature[5,11,23], focus group, expert opinion
I am discouraged from suggesting stopping or reducing medications because I feel physicians would find my recommendations unknowledgeable.	literature[18,22], focus group
I feel I am unable to identify potentially inappropriate medicines a patient could be taking that require stopping or reducing	literature[10], focus group
I feel I do not have enough confidence to suggest stopping or reducing medications.	literature[6,8,10,19,24], focus group, expert opinion
I believe there is a disproportion between available guidelines on prescribing and stopping medications which makes it difficult for me to suggest deprescribing.	literature[6,15,21,22]
I am apprehensive in recommending stopping or reducing preventative medication.	literature[6,13,20], focus group
I do not have enough time to suggest stopping medications on a day-to-day basis.	literature[4,6–9,24], focus group
Stopping or reducing medications requires filling out additional patient documentation which thwarts from suggesting such changes regularly.	focus group
I feel my pharmacy lacks adequate space to counsel patients and suggest changes in pharmacotherapy. //<br>I feel that patient care is fragmented which leads to information loss, and that prevents me from suggesting major changes in patient's pharmacotherapy (such as stopping medications).	literature[8,11,13,18], focus group
Lack of policy and legislation regarding deprescribing makes it difficult for me to suggest stopping or reducing medications.	literature[13,16], focus group, expert opinion
I am willing to suggest deprescribing to my patients if appropriate.	focus group, expert opinion

*Items labelled with * were excluded from the final version of the questionnaire as they did not load into any factors.*

Table S1: Content validity assessment (number of panellist's responses)

	ITEM	essential	useful but not essential	not necessary	CVR
K1	Deprescribing is tapering or reducing a dose of a medication/ is a method of reducing drug dosage.	10	0	0	1
K2	Deprescribing is changing medication to a safer alternative / a method of changing a drug to a safer alternative.	7	3	0	1
K3	Deprescribing is a method of discontinuing a drug without a specific indication or benefit for a patient.	8	2	0	1
A1	Deprescribing medication is as important as prescribing medication.	7	2	1	0,8
A2	Deprescribing reduces health care expenditures/costs.	6	4	0	1
A3	Deprescribing can improve patient adherence.	9	0	1	0,8
A4	Deprescribing improves patient outcomes.	8	1	1	0,8
PF1	If a patient expressed their desire to have the number of their medication reduced, I would suggest stopping medications.	10	0	0	1
PF2	A successful prior stopping of medication would encourage me suggest stopping medications to a patient again.	9	1	0	1
PF3	Easily available patient education materials (brochures, booklets…) would help me educate and suggest stopping medications to my patients more.	5	4	1	0,8
PF4	I am keener to suggest stopping medications to patients who show greater involvement in their medication.	9	0	1	0,8
COLF1	A close collaboration with a pharmacist who could provide patient follow-up during medication withdrawal would encourage me to suggest stopping medications more.	6	4	0	1
COLF2	A close collaboration with a physician to whom I could propose changes in pharmacotherapy would encourage me to suggest stopping medications more.	7	3	0	1
COLF3	I wish physicians would contact pharmacists more often when it comes to patient care.	6	4	0	1
COLF4	Evidence-based rationale behind a pharmacist's suggestion to stop medication would help me accept pharmacist's suggestions.	7	2	1	0,8
COLF5	A public health project on deprescribing including a collaborative model (pharmacist and physicians) would encourage me to suggest deprescribing more.	9	0	1	0,8
COMF1	I feel I need more continuing education on the rationale behind stopping or reducing medication to be able to suggest such changes.	10	0	0	1
COMF2	I feel I need guidelines or algorithms to suggest stopping medications.	9	1	0	1
COMF3	A workshop or webinar on how to approach patients about stopping medication would help me suggest such changes more.	10	0	0	1
COMF4	A course on medication review and medication management would help me suggest stopping or reducing medications more.	8	2	0	1
COMF5	A reminder/decision support tool within healthcare providers software would enable me to suggest stopping medications more.	8	2	0	1
HCSF1	I believe I need to be reimbursed for suggesting stopping medications as I am for dispensing/other clinical services I provide.	8	1	1	0,8
HCSF2	Having the possibility to contact a task force or a professional network when I am having doubts regarding stopping or reducing medications, would encourage me to suggest such changes.	7	2	1	0,8
HCSF3	If I had the possibility to view patients' medical records (or parts of it important to pharmacist), I would be able to suggest stopping or reducing medications more.	9	1	0	1
HCSF4	If I had additional staff members in my practice/pharmacy to take over a part of administrative workload, I would be able to suggest stopping or reducing medications more.	10	0	0	1
PB1	I find it difficult to suggest stopping or reducing medications to patients who have been using them for a long time even if they no longer needed them.	10	0	0	1
PB2	I am concerned that suggesting stopping medications could negatively influence / harm my relationship with my patient.	10	0	0	1
PB3	I find it difficult to suggest stopping or reducing medications to patients who have low understanding of their therapy or to those that have low involvement in medication decision-making.	9	1	0	1

PB4	Patient/patient's caregiver often insist I continue prescribing certain medication even if it is no longer necessary	10	0	0	1
PB5	I worry that stopping medications could lead to adverse drug withdrawal effects or worsening of patient's health.	8	1	1	0,8
COLB1	I believe physicians find it inappropriate for pharmacists to suggest stopping or reducing medications. // I find it inappropriate for pharmacists to suggest stopping or reducing medications to my patients.	8	2	0	1
COLB2	Lack of direct in-real-time communication with other healthcare providers (hospital doctors or specialists, pharmacist, nursing home care teams...) makes it difficult for me to suggest stopping or reducing medications.	7	2	1	0,8
COLB3	I worry that suggesting stopping or reducing medications could negatively influence my relationship with prescribers. // I find it inappropriate to suggest stopping or reducing medications other physicians (other specialists) have prescribed.	9	1	0	1
COLB4	I worry physicians will not understand the rationale behind my suggestions. // I find it inappropriate when another physician suggests stopping medications I have prescribed.	10	0	0	1
COLB5	I am discouraged from suggesting stopping or reducing medications because I feel physicians would find my recommendations unknowledgeable.	8	1	1	0,8
COMB1	I feel I am unable to identify potentially inappropriate medicines a patient could be taking that require stopping or reducing	10	0	0	1
COMB2	I feel I do not have enough confidence to suggest stopping or reducing medications.	10	0	0	1
COMB3	I believe there is a disproportion between available guidelines on prescribing and stopping medications which makes it difficult for me to suggest deprescribing.	8	2	0	1
COMB4	I am apprehensive in recommending stopping or reducing preventative medication.	6	3	1	0,8
HCSB1	I do not have enough time to suggest stopping medications on a day-to-day basis.	5	5	0	1
HCSB2	Stopping or reducing medications requires filling out additional patient documentation which thwarts from suggesting such changes regularly.	6	3	1	0,8
HCSB3	I feel my pharmacy lacks adequate space to counsel patients and suggest changes in pharmacotherapy. // I feel that patient care is fragmented which leads to information loss, and that prevents me from suggesting major changes in patient's pharmacotherapy (such as stopping medications).	6	3	1	0,8
HCSB4	Lack of policy and legislation regarding deprescribing makes it difficult for me to suggest stopping or reducing medications.	8	1	1	0,8
W1	I am willing to suggest deprescribing to my patients if appropriate.	10	0	0	1

CVR is calculated as (Ne – 0.5N)/0.5N, where Ne is number of panellists rating the item as essential or useful, and 0.5N is half the total number of panellists. A total of 10 panellist participated in content validity assessment. Items is considered to have content validity if at least half of panellists assessed it as essential and had the CVR value of at least 0.62.

Supplementary References

1. Scott, I.A.; Hilmer, S.N.; Reeve, E.; Potter, K.; Le Couteur, D.; Rigby, D.; Gnjidic, D.; Del Mar, C.B.; Roughead, E.E.; MClinPharm, A.P.; et al. Reducing inappropriate polypharmacy: The process of deprescribing. *JAMA Intern. Med.* **2015**, *175*, 827–834. https://doi.org/10.1001/JAMAINTERNMED.2015.0324.
2. Reeve, E.; Gnjidic, D.; Long, J.; Hilmer, S. A systematic review of the emerging definition of 'deprescribing' with network analysis: Implications for future research and clinical practice. *Br. J. Clin. Pharmacol.* **2015**, *80*, 1254–1268. https://doi.org/10.1111/BCP.12732.
3. Kua, C.H.; Mak, V.S.L.; Lee, S.W.H. Perspectives of health professionals towards deprescribing practice in Asian nursing homes: A qualitative interview study. *BMJ Open* **2019**, *9*, e030106. https://doi.org/10.1136/bmjopen-2019-030106.
4. Alrasheed, M.M.; Alhawassi, T.M.; Alanazi, A.; Aloudah, N.; Khurshid, F.; Alsultan, M. Knowledge and willingness of physicians about deprescribing among older patients: A qualitative study. *Clin. Interv. Aging* **2018**, *13*, 1401–1408. https://doi.org/10.2147/CIA.S165588.
5. Anderson, K.; Stowasser, D.; Freeman, C.; Scott, I. Prescriber barriers and enablers to minimising potentially inappropriate medications in adults: A systematic review and thematic synthesis. *BMJ Open* **2014**, *4*, e006544. https://doi.org/10.1136/bmjopen-2014-006544.
6. Lundby, C.; Graabæk, T.; Ryg, J.; Søndergaard, J.; Pottegård, A.; Nielsen, D.S. Health care professionals' attitudes towards deprescribing in older patients with limited life expectancy: A systematic review. *Br. J. Clin. Pharmacol.* **2019**, *85*, 868–892. https://doi.org/10.1111/bcp.13861.

7. Mulder-Wildemors, L.G.M.; Heringa, M.; Floor-Schreudering, A.; Jansen, P.A.F.; Bouvy, M.L. Reducing Inappropriate Drug Use in Older Patients by Use of Clinical Decision Support in Community Pharmacy: A Mixed-Methods Evaluation. *Drugs Aging* **2020**, *37*, 115–123. https://doi.org/10.1007/s40266-019-00728-y.
8. Scott, S.; Twigg, M.J.; Clark, A.; Farrow, C.; May, H.; Patel, M.; Bhattacharya, D. Development of a hospital deprescribing implementation framework: A focus group study with geriatricians and pharmacists. *Age Ageing* **2020**, *49*, 102–110. https://doi.org/10.1093/AGEING/AFZ133.
9. Wallis, K.A.; Andrews, A.; Henderson, M. Swimming against the tide: Primary care physicians' views on deprescribing in everyday practice. *Ann. Fam. Med*. **2017**, *15*, 341–346. https://doi.org/10.1370/afm.2094.
10. Bolmsjö, B.B.; Palagyi, A.; Keay, L.; Potter, J.; Lindley, R.I. Factors influencing deprescribing for residents in Advanced Care Facilities: Insights from General Practitioners in Australia and Sweden. *BMC Fam. Pract.* **2016**, *17*, 152. https://doi.org/10.1186/s12875-016-0551-7.
11. Gerlach, N.; Michiels-Corsten, M.; Viniol, A.; Schleef, T.; Junius-Walker, U.; Krause, O.; Donner-Banzhoff, N. Professional roles of general practitioners, community pharmacists and specialist providers in collaborative medication deprescribing—A qualitative study. *BMC Fam. Pract*. **2020**, *21*, 183. https://doi.org/10.1186/s12875-020-01255-1.
12. Nadarajan, K.; Balakrishnan, T.; Yee, M.L.; Soong, J.L. The attitudes and beliefs of doctors towards deprescribing medications. *Proc. Singapore Healthc*. **2018**, *27*, 41–48. https://doi.org/10.1177/2010105817719711.
13. Ailabouni, N.J.; Nishtala, P.S.; Mangin, D.; Tordoff, J.M. Challenges and enablers of deprescribing: A general practitioner perspective. *PLoS ONE* **2016**, *11*, e0151066. https://doi.org/10.1371/journal.pone.0151066.
14. Cross, A.J.; Le, V.J.; George, J.; Woodward, M.C.; Elliott, R.A. Stakeholder perspectives on pharmacist involvement in a memory clinic to review patients' medication management and assist with deprescribing. *Res. Soc. Adm. Pharm*. **2020**, *16*, 681–688. https://doi.org/10.1016/j.sapharm.2019.08.024.
15. Schuling, J.; Gebben, H.; Veehof, L.J.G.; Haaijer-Ruskamp, F.M. Deprescribing medication in very elderly patients with multimorbidity: The view of Dutch GPs. A qualitative study. *BMC Fam. Pract*. **2012**, *13*, 56. https://doi.org/10.1186/1471-2296-13-56.
16. Conklin, J.; Farrell, B.; Suleman, S. Implementing deprescribing guidelines into frontline practice: Barriers and facilitators. *Res. Soc. Adm. Pharm*. **2019**, *15*, 796–800. https://doi.org/10.1016/j.sapharm.2018.08.012.
17. Farrell, B.; Tsang, C.; Raman-Wilms, L.; Irving, H.; Conklin, J.; Pottie, K. What are priorities for deprescribing for elderly patients? Capturing the voice of practitioners: A modified delphi process. *PLoS ONE* **2015**, *10*, e0122246. https://doi.org/10.1371/JOURNAL.PONE.0122246.
18. Kouladjian, L.; Gnjidic, D.; Reeve, E.; Chen, T.F.; Hilmer, S.N. Health Care Practitioners' Perspectives on Deprescribing Anticholinergic and Sedative Medications in Older Adults. *Ann. Pharmacother*. **2016**, *50*, 625–636. https://doi.org/10.1177/1060028016652997.
19. Djatche, L.; Lee, S.; Singer, D.; Hegarty, S.E.; Lombardi, M.; Maio, V. How confident are physicians in deprescribing for the elderly and what barriers prevent deprescribing? *J. Clin. Pharm. Ther*. **2018**, *43*, 550–555. https://doi.org/10.1111/jcpt.12688.
20. Luymes, C.H.; van der Kleij, R.M.J.J.; Poortvliet, R.K.E.; de Ruijter, W.; Reis, R.; Numans, M.E. Deprescribing Potentially Inappropriate Preventive Cardiovascular Medication: Barriers and Enablers for Patients and General Practitioners. *Ann. Pharmacother*. **2016**, *50*, 446–454. https://doi.org/10.1177/1060028016637181.
21. Cullinan, S.; Raae Hansen, C.; Byrne, S.; O'Mahony, D.; Kearney, P.; Sahm, L. Challenges of deprescribing in the multimorbid patient. *Eur. J. Hosp. Pharm*. **2017**, *24*, 43–46. https://doi.org/10.1136/ejhpharm-2016-000921.
22. Anderson, K.; Foster, M.; Freeman, C.; Luetsch, K.; Scott, I. Negotiating "Unmeasurable Harm and Benefit": Perspectives of General Practitioners and Consultant Pharmacists on Deprescribing in the Primary Care Setting. *Qual. Health Res*. **2017**, *27*, 1936–1947. https://doi.org/10.1177/1049732316687732.
23. Baysari, M.T.; Duong, M.; Zheng, W.Y.; Nguyen, A.; Lo, S.; Ng, B.; Ritchie, A.; Le Couteur, D.; McLachlan, A.; Bennett, A.; et al. Delivering the right information to the right person at the right time to facilitate deprescribing in hospital: A mixed methods multisite study to inform decision support design in Australia. *BMJ Open* **2019**, *9*, 30950. https://doi.org/10.1136/bmjopen-2019-030950.
24. Rossi, P.R.; Hegarty, S.E.; Maio, V.; Lombardi, M.; Pizzini, A.; Mozzone, A.; Uberti, M.; Miozzo, S. General practitioner attitudes and confidence to deprescribing for elderly patients. *Geriatr Care*. **2020**, *6*. https://doi.org/10.4081/gc.2020.8703.

Table S2: Distribution of factor scores

	pharmacists' version				physicians' version			
**factor**	min	max	mean	std.dev.	min	max	mean	std.dev.
knowledge	1.00	5.00	4.04	0.88	1.67	5.00	3.71	0.84
awareness	1.00	5.00	4.57	0.57	1.25	5.00	4.19	0.77
patient facilitator	1.00	5.00	3.63	0.81	2.00	5.00	3.77	0.67
collaboration facilitator	1.00	5.00	4.51	0.58	1.00	5.00	3.78	0.93
competences facilitator	1.00	5.00	4.45	0.65	1.25	5.00	3.95	0.84
healthcare system facilitator	1.00	5.00	4.22	0.78	1.00	5.00	3.90	0.80
patient barrier	1.00	4.75	3.21	0.72	1.00	4.75	3.11	0.81
collaboration barrier	1.00	5.00	3.65	1.01	1.50	4.75	3.16	0.76
competences barrier	1.00	5.00	3.42	0.74	1.00	5.00	2.87	0.82
healthcare system barrier	1.00	5.00	3.89	0.75	1.75	5.00	3.84	0.81

Table S3 Test-retest participants characteristics

Characteristics	
Sex female (n,%)	17 (85%)
Age (median, IQR)	33 (26-40)
Professional experience (median, IQR)	7(2-14)
Practice location (n,%)	
urban	14 (70%)
suburban	4 (20%)
rural	2 (10%)
Practice placement (n,%)	
within another healthcare facility	3 (15%)
near another healthcare facility	5 (25%)
within a shopping facility	1 (5%)
displaced (not near any facility)	11 (55%)
Patient population (median, IQR)	
number of patients	1880 (1770-1940)
percentage of elderly patients (>65 years)	55% (50-66)
