# Supplementary material for: Development and Validation of Comprehensive Healthcare Providers’ Opinions, Preferences, and Attitudes towards Deprescribing (CHOPPED Questionnaire)
Source: Pharmacy (Basel). 2022 Jul 1;10(4):76. doi: 10.3390/pharmacy10040076 (PMC9326567; doi:10.3390/pharmacy10040076)
Supplement: Supplementary file 1 [file pharmacy-10-00076-s001.zip › file S2.pdf]

Development and Validation of Comprehensive Healthcare Providers’ Opinions, Preferences, and Attitudes towards Deprescribing (CHOPPED Questionnaire)

Iva Bužančić and Maja Ortner-Hadžiabdić

File S2: CHOPPED case vignette

A well-known, long-term patient of yours, Mr. X., is complaining about the number of medications he is taking. He would like to reduce the number of medication and pills he takes daily. His basic medical information states the following: 76 years, 178 cm/70 kg/BMI 22.09, waist circumference 80 cm.

He complains about the following symptoms: often feeling dizzy, especially in the morning and when standing up; throughout the day, he feels tired and sleepy and has a feeling he is starting to forget more often. He experienced two falls withing the last six months, with minor bruising. Due to dietary changes, he noticed a 5–6 kg weight loss within the last year. His extremities do not present oedema.

The latest laboratory values of electrolytes, kidney, and liver functions are within the range for the patient’s age (eGFR 80 ml/min/1.73m2; total cholesterol 5.1 mmol/L; HDL 1.6 mmol/L; LDL 2.8 mmol/L; triglycerides 1.3 mmol/L).

Arterial hypertension (had for longer than 20 years, takes medication regularly, HBPM 105–115/75 mmHg throughout the last year. Positive family history of hypertension, but negative for CV incidents).

Furosemide 40 mg 1x daily in the mornings, **moxonidine 0.4 mg** 1x daily in the evening—5 years.

Amlodipine/valsartan 10mg/160 mg 1x daily in the mornings, bisoprolol 5 mg 1x daily in the afternoon—15 years.

Gout (had for around 10 years):

Allopurinol 200 mg 1x daily introduced five years ago after an acute exacerbation, until then the condition was regulated with dietary measures.

Ibuprofen 600 mg 1–3x daily taken daily for the past two years since the last exacerbation.

Tramadol/dexketoprofen 75 mg/25 mg 1–2x daily—introduced two years ago to treat an acute exacerbation at the time, prescribed by a physiatrist.

Anxiety disorder (diagnosed three years ago, after losing his spouse). All medication taken regularly, i.e., daily for the past three years.

Alprazolam 0.5 mg 1-3x daily, zolpidem 10 mg 1x daily, and **escitalopram 10 mg** 1x daily.

Benign prostatic hyperplasia prostate and overactive bladder; regular urologist visits (PSA low and within range): **tamsulozin 0.4 mg** 1x daily (four years ago); solifenacin 10 mg 1x daily (one year ago).

Additional pharmacotherapy:

Pantoprazole 40 mg 2x daily—started two years ago;

Multivitamin supplement 1x daily—for the past three years;

Acetylsalicylic acid 100 mg 1x daily—for the past 7 years;

Lactulose 667mg/ml 15–30 ml 2–3x a week for the past year.

PHARMACISTS' VERSION:

Which medication/medications would you suggest deprescribing to the patient and the prescriber? Please state your rationale/evidence for your choice/choices.

a)	moxonidine	g)	solifenacin	m)	bisoprolol
b)	furosemide	h)	acetylsalicylic acid	n)	amlodipine/valsartan
c)	NSAID	i)	allopurinol	o)	escitalopram
d)	opioid	j)	pantoprazole	p)	multivitamin
e)	alprazolam	k)	tamsulozin	q)	no medication
f)	zolpidem	l)	lactulose		

PHYSICIANS' VERSION:

With which pharmacists deprescribing suggestion and rationale would you agree?

a) Moxonidine: reduce the dose; if appropriate, discontinue medication if BP levels are satisfactory; centrally acting antihypertensives may increase the risk of falls, sedation, and constipation; may increase the sedative effect of anxiolytics and sedatives [1].

b) Furosemide: reduce the dose, discontinue if BP levels are satisfactory; diuretics are a compelling contraindication in patients with gout; increased risk of orthostatic hypotension; BP control can be achieved by increasing the dose of ARB+CCBa; should be prescribed in the case of oedema [2].

c) NSAID: reduce the dose; it is contraindicated to use two NSAIDs at the same time; possible clinically significant interactions (NSAID–antihypertensives and NSAID–SSRI) and increased risk of GIT bleeding; renal damage; loss of antihypertensive effect; and increased risk of cardiovascular adverse events [3].

d) Opioid: limit use to eight weeks, not recommended to be used with other CNS depressors; increased risk of falls; anticholinergic side effects [4].

e) Alprazolam: limit use to 4–12 weeks for anxiety treatment (initiate treatment with antidepressant); long-term use linked to unfavorable outcomes such as falls, cognitive problems, and daytime tiredness [5].

f) Zolpidem: reduce the dose; if possible, discontinue medication entirely, as long-term use of hypnotics is linked to unfavorable outcomes such as falls, cognitive problems, and daytime tiredness [5].

g) Solifenacin: decrease the dose in the case of anticholinergic side effects such as constipation, dry mouth, blurred vision, and drowsiness [6].

h) Acetylsalicylic acid: lack of indication for primary prevention.

i) Allopurinol: decreasing and discontinuing diuretics alongside with dietary changes might lower urate levels; consider decreasing the dose [7].

j) Pantoprazole: not recommended beyond 12 weeks unless used by patients with high GIT bleeding risk; reduce the dose and dosing; recommend using when NSAID is used; use H_2 antagonists if necessary; prolonged use my lead to increased risk of fractures, vitamin B12 deficiency, and C. difficile infections [8].

l) Lactulose: lack of indication or continuous use unless constipation is cause by use of opioids and solifenacin.

p) Multivitamin supplement.

q) No medication.

Supplementary References

1. Primary health Tasmania. Deprescribing resources. Antihypertensives. Available: https://www.primaryhealthtas.com.au/resources/deprescribing-resources/ (Accessed on 20 December 2021)
2. Eureopan Society of Cardiology. Management of Arterial hypertension. https://www.escardio.org/Guidelines/Clinical-Practice-Guidelines/Arterial-Hypertension-Management-of (Accessed on 20 December 2021)
3. Primary health Tasmania. Deprescribing resource. NSAIDS. https://www.primaryhealthtas.com.au/wp-content/uploads/2018/09/A-Guide-to-Deprescribing-NSAIDS-2019.pdf (Accessed on 20 December 2021)
4. Primary health Tasmania. Deprescribing resource. Opioids. https://www.primaryhealthtas.com.au/wp-content/uploads/2018/09/A-Guide-to-Deprescribing-Opioids-2019.pdf (Accessed on 20 December 2021)
5. Pottie, K.; Thompson,W.; Davies, S.; Grenier, J.; Sadowski, C.A.; Welch, V.; Holbrook, A.; Boyd, C.; Swenson, R.; Ma A.; Farrell, B. Deprescribing benzodiazepine receptor agonists: Evidence-based clinical practice guideline. Can Fam Physician. 2018 May;64(5):339-351. https://deprescribing.org/wp-content/uploads/2019/03/deprescribing_algorithms2019_BZRA_vf-locked.pdf
6. NSW Theraupeutic Advisory Group Inc. Deprescribing tools. Deprescribing gude for Genitourinary drus. https://www.nswtag.org.au/deprescribing-tools/ (Accessed on 20 December 2021)
7. Primary health Tasmania. Deprescribing resource. Allopurninol. https://www.primaryhealthtas.com.au/wp-content/uploads/2018/09/A-Guide-to-Deprescribing-Allopurinol-2019.pdf (Accessed on 20 December 2021)
8. Farrell, B.; Pottie, K.; Thompson, W.; Boghossian, T.; Piazzola, L.; Rashid, F.J, et al. Deprescribing proton pump inhibitors. Evidence-based clinical practice guideline. Can FAm Physician 2017; 63:354-64 https://deprescribing.org/wp-content/uploads/2018/08/ppi-deprescribing-algorithm_2018_En.pdf
